# Supplementary material for: Assessment of simulations of a polar low with the Canadian Regional Climate Model
Source: PLoS One. 2023 Oct 5;18(10):e0292250. doi: 10.1371/journal.pone.0292250 (PMC10553340; doi:10.1371/journal.pone.0292250)
Supplement: Attachment — Submitted filename: Response to Reviewers.pdf [file pone.0292250.s003.pdf]

Response to the reviewer

Thank you very much for your valuable comments, which have helped improve the manuscript. Following your suggestion, we have deleted the sentences in Lines 11-15.

Response to the journal

Following the suggestion of the reviewer, we have deleted the sentences in Line 11-15.
We have also reviewed our reference list to ensure that it is complete and correct.

We have uploaded our figure files to the Preflight Analysis and Conversion Engine (PACE) digital diagnostic tool to ensure that our figures meet PLOS requirements.
