# Supplementary material for: Assessment of simulations of a polar low with the Canadian Regional Climate Model
Source: PLoS One. 2023 Oct 5;18(10):e0292250. doi: 10.1371/journal.pone.0292250 (PMC10553340; doi:10.1371/journal.pone.0292250)
Supplement: Attachment — Submitted filename: COMMENT-PONE-D-22-29970.docx [file pone.0292250.s001.docx]

Polar lows (PL) are always associated with severe weather conditions and its forecasting remains a challenge. This study focus on the impact of the initial conditions of the simulation of PL using the convection-permitting model of CRCM6/GEM4. This study shows some new phenomena. However, the physical mechanism behind the phenomenon is not explained here. Thus, the followings need to be commented or addressed before it is publishable.

**General comments**

1. This article is mainly focus on the description of phenomena, but without the exploration of physical mechanism. The author should give a discussion about the possible reason leading to the difference among the simulations.
2. ERA5 has more skill than the CPM simulation in producing the PL. This conclusion maybe related with the choice of parameterization schemes. Whether the authors have compared the simulations among the different parameterization schemes.
3. They compared the simulation with their driving forcing of ERA5 and found that the CPM does not seem to provide much added value compared to ERA5. However, they didn’t calculate the added value. I recommend that the authors should evaluate the simulation improvements with downscaling using the method proposed by Di Luca et al. (2013).

Di Luca A, Elía R, Laprise R. 2013. Potential for small scale added value of RCMs downscaled climate change signal. Clim. Dyn. 40: 601–618.

specific comments

1. Some of the sentence in this study may be result in confusion. For example, Line 26-29.
2. To much lines in figure 4, and it is difficult to identify.
