# Supplementary material for: Assessment of simulations of a polar low with the Canadian Regional Climate Model
Source: PLoS One. 2023 Oct 5;18(10):e0292250. doi: 10.1371/journal.pone.0292250 (PMC10553340; doi:10.1371/journal.pone.0292250)
Supplement: Attachment — Submitted filename: Response to Reviewers.docx [file pone.0292250.s002.docx]

**RESPONSE TO REVIEWERS**

Thank you very much for your constructive comments, which have helped improve the manuscript.

**General comments**

1. **This article is mainly focus on the description of phenomena, but without the exploration of physical mechanism. The author should give a discussion about the possible reason leading to the difference among the simulations.**

A new section (“3.4 Final discussion”, p.30-32) has been added to the manuscript to discuss the reasons that lead to the difference between the simulations. This section includes three new figures (12, 13, 14) to support the discussion. Several sentences have also been added to the abstract and the conclusion section to include this analysis.

2. **ERA5 has more skill than the CPM simulation in producing the PL. This conclusion maybe related with the choice of parameterization schemes. Whether the authors have compared the simulations among the different parameterization schemes.**

We have not conducted simulations using different parameterization schemes since the objective of our study was to quantify the impact of the initial conditions on the simulation of the PL, so our simulations only differ in the initial conditions.

In the “Conclusion” section, we suggest that one of the main reasons that could explain the fact that ERA5 has better skill than CRMC6/GEM4 is that conventional observations are assimilated into ERA5. Other plausible reasons for the different performance are the differences between the forecast model used in the reanalysis and the CRMC6/GEM4 (e.g., the different parameterizations, as the reviewer points out). Accordingly, in the “Conclusion” section, we mention that a new version of GEM with improved physics parameterizations was recently released and suggest that an interesting course of research would be to analyse if this new version of GEM provides added value compared to ERA5.

3. **They compared the simulation with their driving forcing of ERA5 and found that the CPM does not seem to provide much added value compared to ERA5. However, they didn’t calculate the added value. I recommend that the authors should evaluate the simulation improvements with downscaling using the method proposed by Di Luca et al. (2013).**

**Di Luca A, Elía R, Laprise R. 2013. Potential for small scale added value of RCMs downscaled climate change signal. Clim. Dyn. 40: 601–618**.

According to Di Luca et al. (2013; equation 1), if we use the squared error (SE) as metric to evaluate the model performance, the added value (AV) can be defined as

$$AV={SE}_{GCM}-{SE}_{RCM}$$

where *X* is some climate statistics (e.g., seasonal-averaged precipitation), GCM is the lower resolution climate model and RCM is a regional climate model. In our study, the lower resolution dataset is ERA5 reanalysis and, using the root mean squared error (RMSE) to evaluate the model performance, the added value is

$$AV={RMSE}_{ERA5}-{RMSE}_{RCM}$$

If we take *X* as the aggregate statistics, then the added value can be easily computed from the information provided in Tables 2-8. Following your suggestion, we have added Table 9, which includes the added value of the best simulation compared to ERA5. Table 9 and a few sentences discussing the results shown therein have been included in the “Conclusion” (section 4).

**Specific comments**

1. **Some of the sentence in this study may be result in confusion. For example, Line 26-29.**

We have modified lines 25-29 of the original manuscript as follows:

“The polar regions experience a variety of climate-related extreme events and high-impact weather conditions such as katabatic winds, blizzards, and polar lows (PLs) [1]. PLs are intense mesoscale maritime cyclones that develop between the poles and the main baroclinic zone, mainly during the cold season.”

2. **To much lines in figure 4, and it is difficult to identify.**

Figure 4 has been modified to make it clearer, and the legend has been modified accordingly.
